# Reproductive phenology is a repeatable, heritable trait linked to the timing of other life-history events in a migratory marine predator

**DOI:** 10.1098/rspb.2023.1170

**Published:** 2023-07-26

**Authors:** W. C. Oosthuizen, P. A. Pistorius, M. N. Bester, R. Altwegg, P. J. N. de Bruyn

**Affiliations:** ^1^ Centre for Statistics in Ecology, Environment and Conservation, Department of Statistical Sciences, University of Cape Town, Cape Town 7701, South Africa; ^2^ Marine Apex Predator Research Unit, Institute for Coastal and Marine Research and Department of Zoology, Nelson Mandela University, Gqeberha 6031, South Africa; ^3^ Mammal Research Institute, Department of Zoology and Entomology, University of Pretoria, Private Bag X20, Hatfield, Pretoria 0028, South Africa

**Keywords:** arrival date, breeding timing, *Mirounga leonina*, phenology, phenotypic plasticity, repeatability

## Abstract

Population-level shifts in reproductive phenology in response to environmental change are common, but whether individual-level responses are modified by demographic and genetic factors remains less well understood. We used mixed models to quantify how reproductive timing varied across 1772 female southern elephant seals (*Mirounga leonina*) breeding at Marion Island in the Southern Ocean (1989–2019), and to identify the factors that correlate with phenological shifts within and between individuals. We found strong support for covariation in the timing of breeding arrival dates and the timing of the preceding moult. Breeding arrival dates were more repeatable at the individual level, as compared with the population level, even after accounting for individual traits (wean date as a pup, age and breeding experience) associated with phenological variability. Mother–daughter similarities in breeding phenology were also evident, indicating that additive genetic effects may contribute to between-individual variation in breeding phenology. Over 30 years, elephant seal phenology did not change towards earlier or later dates, and we found no correlation between annual fluctuations in phenology and indices of environmental variation. Our results show how maternal genetic (or non-genetic) effects, individual traits and linkages between cyclical life-history events can drive within- and between-individual variation in reproductive phenology.

## 1. Introduction

Long-term data from terrestrial, fresh-water and marine organisms have revealed significant plasticity in the timing (phenology) of seasonal life-cycle events in response to environmental change. For example, long-term warming trends have advanced spring events such as flowering, migration and breeding in many plants and animals [1]. Altered growing and breeding seasons, and disruptions to interactions within and among species, may have significant consequences for individual fitness, population dynamics and ecosystem resilience [2]. Phenological responses to environmental change vary greatly across taxonomic, trophic and regional groups, but long-term phenological datasets are biased towards plants, arthropods and birds from Northern Hemisphere temperate regions [1,3]. Moreover, the mechanisms that underpin patterns of variation in phenological responses often remain unknown [4], limiting our capacity to predict future patterns of phenological change in populations that can potentially have cascading effects on communities and ecosystems globally.

Population-level phenological change in life-history events (e.g. birth or egg-laying dates) arises from individual responses to changing environmental conditions, and/or via changes in population structure that may then shape trait distributions [5]. We can thus improve our understanding of changes in phenology by quantifying the amount of variance attributed to within-individual and between-individual effects [6]. Mechanisms responsible for phenological change include adaptive evolution (changes in gene frequencies due to selection [7]), phenotypic plasticity (environmentally dependent gene expression [8,9]) and demography (age-dependent phenological variation [10]). The timing of birth in mammals, for example, often tracks environmental fluctuations (annual changes in precipitation, vegetation phenology or population density) through plastic, demographic and genetic mechanisms [7,11]. Failure to track environmental change can lead to a mismatch between resources and birth dates, which may in turn reduce fitness [12].

Although animals might exhibit phenotypic plasticity (responsiveness to changing environments) at the within-individual level, there often remain consistent between-individual differences in average phenology, even after environmental and demographic factors are accounted for. One explanation for consistent between-individual differences is that phenotypic flexibility within individuals is constrained by linkages between cyclical life-history events (e.g. migration and reproduction, or moult and reproduction). Carry-over effects between different phenological events may therefore limit phenotypic flexibility within individuals [13,14]. Another possibility is that between-individual phenotypic plasticity is determined by additive genetic effects (e.g. heritability of egg-laying or parturition dates [15]) that are maintained across environmental or demographic contexts. Both temporal and between-individual variation in the timing of reproduction can have important consequences for reproductive success and consequently for population dynamics [16,17]. It is therefore important to understand how reproductive phenology differs within and between individuals, and whether individuals and populations express repeatability and/or phenotypic plasticity in the timing of reproduction.

Southern elephant seals (*Mirounga leonina*; hereafter elephant seals) are long-lived Southern Ocean marine mammals that breed and moult on land. Female elephant seals maintain highly synchronized seasonal reproduction, probabaly through photoperiodic cueing but also through variables in addition to daylength (e.g. environmental conditions) [18,19] (electronic supplementary material, text S1 and figure S1). At the individual level, the timing of reproduction varies by age, breeding experience, and the timing of preceding life-cycle events (moult phenology) [20–25]. Foundational studies [20–25] laid important groundwork but were limited by the longitudinal data available at the time. For example, age effects and individual repeatability in breeding phenology have not been studied over the lifetime of individuals, and the extent of phenotypic variation both within and between individuals remains unknown. Furthermore, it is not known whether environmental forcing (e.g. rapid warming of the Southern Ocean and a trend toward the positive phase of the Southern Annular Mode (SAM) [26,27]) is impacting the breeding phenology of elephant seals.

The aim of this study was to quantify the variability in reproductive phenology within and between individually marked female elephant seals breeding at Marion Island in the Southern Ocean over 30 years (1989–2019). We estimated the repeatability (consistency) of arrival dates at the breeding colony across individuals and years and tested the hypotheses that timing of arrival is the result of: (1) individual traits (wean date as a pup, breeding age and breeding experience); (2) environmental conditions (long-term phenological shifts, population density and climate indices); (3) covariation between life-cycle events (linkages between moult and breeding phenology); and (4) narrow-sense heritability. Our results not only describe, for the first time to our knowledge, how additive genetic effects and early-life traits correlate with highly repeatable reproductive phenology in elephant seals, but also extend the existing literature on age effects and linkages between life-cycle events by analysing long-term data (that include individuals across the age spectrum) in a behavioural reaction norm framework.

## 2. Material and methods

### (a) Study species, field methods and data

**Figure 1.**
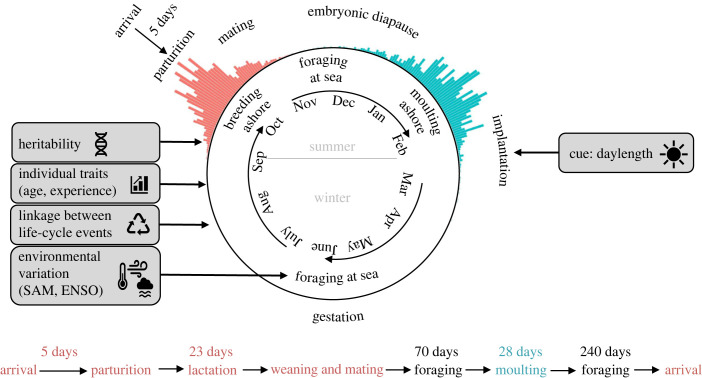
Annual cycle of adult female southern elephant seals. Two major at-sea migrations separate the breeding season (red histogram of arrival dates at Marion Island) in the austral spring and the moulting season (blue histogram of departure dates from Marion Island) in the summer. This study estimated the repeatability of breeding season arrival dates and tested whether arrival timing is influenced by environmental conditions (SAM, Southern Annular Mode; ENSO, El Niño-Southern Oscillation), covariation between life-cycle events, individual traits and narrow-sense heritability.

The annual cycle of adult female elephant seals is characterized by two major haulout events (breeding and moulting) separated by foraging migrations over several thousand kilometres (figure 1). Between September and November each year, elephant seal females (aged three and older) give birth to a single pup 5 days after arriving at the breeding colony [21,28,29]. Female elephant seals are capital breeders that remain ashore continuously without feeding for approximately four weeks; their metabolic energy is obtained from catabolism of blubber lipids and body protein [20]. Because females are depleting finite energy reserves while hauled out, there is likely strong selective pressure to have a short pre-partum period ashore, and thus to precisely time their arrival at the breeding colony. An internal map sense (awareness of geographic location) allows females to adjust their at-sea migrations to give birth within days of their arrival at the colony [30]. Arrival at the breeding colony and timing of birth is therefore more strongly coupled in elephant seals than many other migratory species, where post-migration establishment of a territory, courtship and mating may induce variable lags between arrival and birth or egg-laying dates. Females become sexually receptive and copulate when their pups are close to weaning. Although fertilization takes place at this time, implantation of the blastocyst only occurs towards the end of the summer moult, after an embryonic diapause of about four and a half months [20,31]. All elephant seals older than pups moult ashore annually for a month or more from November to February. Juvenile seals moult progressively later with age, but adult breeding females aged four and older have similar moult starting dates [32,33]. After the moult, adults return to sea to forage up until the next breeding season (figure 1).

From 1983 to 2019, nearly all elephant seal pups born at Marion Island were uniquely marked with two hind-flipper tags (*n* = 8985 female pups) [34,35]. The pups were marked at the first encounter after weaning, after their mothers had abandoned them. However, since 2006, we have also marked some pre-weaned pups with ancillary tags [36], thereby collecting pedigree information on 2115 mother–daughter pairs.

We analysed 30 years (1989–2019) of observations of individually-identified, breeding female elephant seals. We excluded a single breeding season (1998) from analysis owing to poor observation effort. In other years, there was a high probability of identifying individual breeding and moulting females, though not all seals were sighted at every survey [37] (electronic supplementary material, text S2 and figure S2). Tag-resight surveys were made on a daily or near-daily basis in the breeding season, but because elephant seals haul out along more than 50 km of coastline that is only accessible on foot, every specific breeding site was generally only surveyed once every 7 days. We defined each individual's arrival date at the breeding colony as the earliest observation in a particular year. The arrival dates of females at the breeding colony (response variable) and two predictor variables (weaning dates of pups and moult departure dates, described below) thus incorporate measurement error—a point we return to in the discussion. Non-breeding females (pre-breeders and mature females skipping reproduction) may attend breeding aggregations but often remain at sea [38]. We excluded records of females that were not seen with a pup, and breeding females that were not seen in the preceding moult, from analysis.

### (b) Quantifying individual variation with mixed models

We used linear mixed models to partition variation in arrival dates into within- and between-individual variance components, and to test hypotheses about the effects that predictor variables have on the mean of the response variable. Analyses were conducted in R (v4.1.3) [39] using the *lme4* [40] package. The breeding season arrival date (Julian date) of female *i* in year *j* was modelled as a function of several fixed (population-level) and random effects. The following variables were used to describe the fixed-effect component of the model.

#### (i) Linking arrival date to individual traits

We fitted the factors breeding state (first-time breeder or experienced breeder) and female age, the interaction between age and breeding state, and females' Julian date of weaning as a pup (continuous variable) as fixed effects to investigate the influence of individual traits on arrival date. The ages of all females were known because all were tagged as weaned pups, and breeding state could be assigned with confidence given the high monitoring effort. The weaning date covariate, a proxy for birth date, was used to test the prediction that female pups that were born early in the breeding season also breed earlier as adults, and *vice versa*.

#### (ii) Covariation between life-cycle events

To assess covariation between breeding and moulting phenology [25], the Julian date of the last moult observation during the preceding summer was fitted as a fixed-effect covariate (this variable was also fitted as a reaction norm slope—see below).

#### (iii) Linking arrival date to environmental variation

To test hypotheses about environmental influences on phenology we fitted year, population density and climatic variables as linear continuous covariates (additional information and time-series figures of environmental covariates are given in the electronic supplementary material, text S3 and figures S4–S6). We used the *z*-standardized total breeding female population size on 15 October each year as a proxy of population density. For climate-driven environmental variation, we calculated annual means of the Southern Oscillation Index (SOI) and Southern Annular Mode (SAM) from monthly data (during the pre-breeding period, March to September) [41]. These indices quantify environmental conditions at a large scale, are known to widely affect marine predator populations within the Southern Ocean, and may better predict ecological processes compared with local weather indices [42]. Because all individuals did not experience the same set of climatic conditions, we used within-subject centring [43] to determine whether climatic covariates explained a significant amount of either the within-individual or the between-individual variation in the response variable. This method divides the effect of a covariate *x* into two terms (the mean covariate value for all observations of an individual *i* ($\bar{x_{i}}$) and the deviation from the mean for each observation *k* ($x_{ik}-\;\bar{x_{i}}$)). The two terms (instead of$\;x_{ik}$) are then fitted as fixed effects in the model [44].

All models were fitted with random effects for female identity and year. Year was fitted as a random intercept to model any annual variation in mean arrival dates that is not explained by the fixed effects. The female identity random intercept allowed individuals to deviate from the population mean arrival date, while we also considered individual variance in phenotypic plasticity (i.e. deviation from the population average slope) via models that specified trait–environment [45] and trait–trait [46] reaction norm slopes. The random intercepts, assumed to be normally distributed with a mean of zero and a variance *σ*, partitioned the total phenotypic variance ($\sigma_{P}^{2}$) in arrival dates into three levels of interest: between-individual ($\sigma_{i}^{2}$), between-year ($\sigma_{j}^{2}$), and residual (within-individual) ($\sigma_{\varepsilon }^{2}$) variance components [47]. Using package *rptR* [48] in R, we calculated repeatability (*R*), a variance-standardized estimate of the magnitude and consistency of individual (and annual) differences in arrival dates. Repeatability is the ratio of the between-individual ($\sigma_{i}^{2}$) or between-year ($\sigma_{j}^{2})$ variance and the total phenotypic variance$\;\sigma_{P}^{2}$ [48]. Repeatability was calculated from the most parsimonious linear mixed model, including fixed effects, but the variance explained by fixed effects was added back into the total variance before calculating repeatability [49]. Confidence intervals (CI) for repeatability were estimated using parametric bootstrapping (using default *rptR* settings [48]).

### (c) Model building and selection

The first step in our analysis was to test for individual plasticity in trait–trait and trait–environment reaction norms. We set up mixed models with random intercepts for year and female identity, and random slopes for moult date (trait–trait reaction norm), population density and climatic variables (the within-subject mean slopes for population density, SOI and SAM). We used likelihood ratio tests (−2 times the difference in log likelihood distributed as *χ*^2^ with 2 degrees of freedom given that an intercept–slope covariance was estimated in all models) to compare models with random slope terms with models that assumed constant slopes. The random effect structure of the best-fitting model was retained to determine the optimal fixed-effect structure. We used Akaike's information criterion (AIC) to rank models with different fixed effects based on parsimony [50]. Models with the lowest AIC values represent the best compromise between model complexity (parameter count) and model fit (lower deviance), and model parsimony worsens gradually as ΔAIC (the difference between the model with the lowest AIC score and the model in question) increases. The most parsimonious model identified was refitted using restricted maximum likelihood (REML) to obtain parameter estimates. Diagnostic analysis and plots of model residuals did not indicate strong violations of model assumptions (electronic supplementary material, table S1 and figures S7 and S8).

### (d) Hereditary variation in arrival date

We identified 216 instances within our dataset in which we had information on breeding arrival dates of both mothers and their daughters when recruited into the adult population. This subsample was used to examine narrow-sense heritability in arrival date (i.e. the proportion of phenotypic variance that is due to additive genetic values) [51]. The data were not detailed enough to build pedigrees that would allow the genetic basis of phenological variation to be determined with ‘animal models’ [52]. We therefore used parent–offspring regression to estimate narrow-sense heritability (*h^2^*) in breeding season arrival date. The arrival date of first-time breeding daughters was regressed against the arrival date of their mothers when they first reproduced. In this case, narrow-sense heritability is twice the slope of the single-parent regression [53]. When arrival dates were known for mothers and more than one daughter, we used the daughters' mean arrival date, which reduced the data to 187 dyads [53]. A permutation test was used to estimate the probability of obtaining the observed mother–daughter heritability estimate by chance. We constructed 10 000 simulated datasets where the association pairs were not mothers and daughters, but instead dyads drawn at random from the population. We expected the heritability estimate from mother–daughter pairs to be more extreme than that of dyads drawn at random. To obtain a one-tailed significance statistic (*p*-value) we counted how many of the permuted datasets had heritability estimates larger than the one we observed in the actual data.

## 3. Results

Our final dataset included 1772 females breeding 5297 times between 1989 and 2019. Females bred in 5.45 breeding seasons, on average (s.d. = 3.46, range = 1 to 16). Breeding season arrival dates ranged from early September through to November, although 80% of females first came ashore between 23 September and 19 October. The median arrival date over the course of our 30-year study was 4 October (electronic supplementary material, figure S3). We found evidence for significant individual plasticity in breeding phenology with respect to moult date, i.e. the slope of the relationship between arrival at the breeding site and the timing of previous moult differed between individuals (likelihood ratio test *χ*^2^ = 16.80, d.f. = 2, *p* < 0.001) (electronic supplementary material, table S2). By contrast, models with random slope terms for environmental variables were hampered by singularity (i.e. the random effects covariance matrices of the models were (close to) zero), and did not improve model fit significantly (electronic supplementary material, table S2).

**Figure 2.**
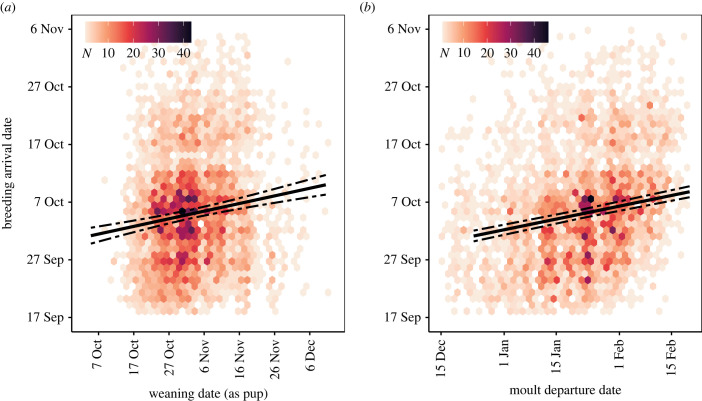
Breeding arrival dates of southern elephant seal females as a function of (*a*) weaning date as a pup and (*b*) the preceding moult haulout departure date. The significant positive linear regression slopes (solid lines, with 95% CI as dashed lines) show that females with earlier weaning dates as pups (*a*) and earlier moult departure dates (*b*) also tended towards earlier breeding arrival dates. We used geom_hex() in ggplot2 [54] to bin scatterplot points into regular hexagons; the distribution of the raw data is thus represented as a two-dimensional heat map of point counts per hexagon (*N*). Figure 3 gives the individual reaction norms related to moult departure date.

**Figure 3.**
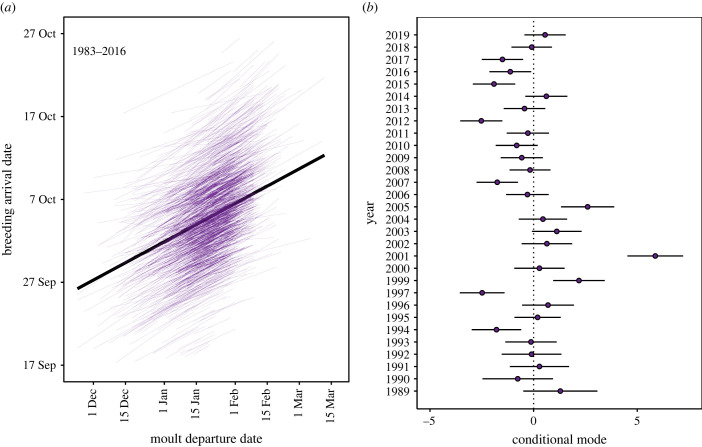
Plotting random intercepts and slopes. (*a*) Influence of the preceding moult departure date on the timing of breeding arrival dates of southern elephant seal females. The thin purple lines are the individual linear reaction norms, i.e. the across-year within-individual change in breeding arrival date as a function of a change in moult date. Individual deviations from the population intercept were larger (standard deviation *σ* = 6.5) and explained more of the variance in the data than individual differences in slopes (plasticity) (*σ* = 1.6). The population-level effect (the average change in breeding arrival date in response to moult date) is given by the thick black regression line. We plot random intercepts and slopes by birth cohorts in the electronic supplementary material, figure S9. (*b*) Predicted annual deviation (means with 95% CI) in breeding arrival dates from the long-term (1989–2019) population-mean value (dashed line).

**Figure 4.**
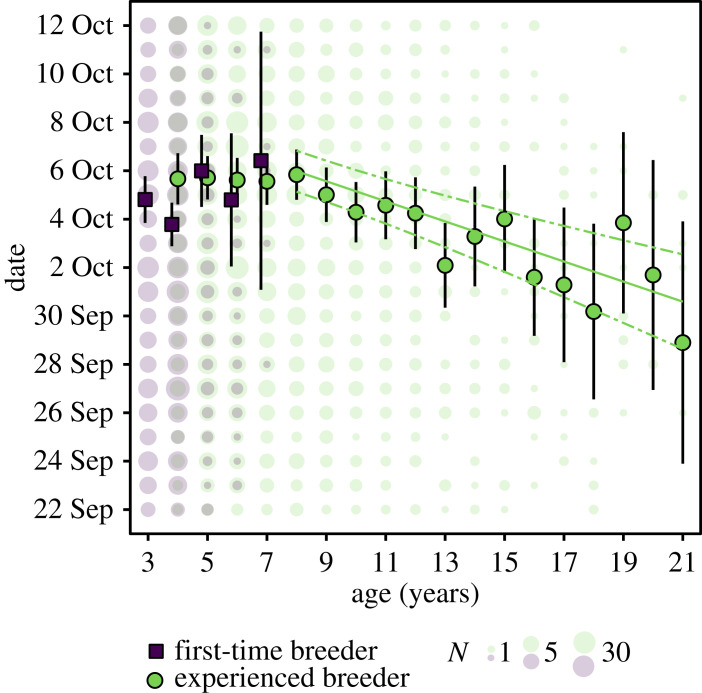
Age- and state- (first-time versus experienced breeders) dependent variation in breeding arrival dates of female southern elephant seals. The observed data, truncated to the limits of this figure, are plotted as circles that scale according to the number of observations. Point estimates (mean arrival dates with 95% CI) were obtained from the model with the lowest AIC score (table 1). The regression line (with 95% CI) from age 8 to age 21 indicates a significant negative linear trend (slope = −0.43 (95% CI: −0.57 to −0.29)) between age and arrival date among older females (electronic supplementary material, text S4).

Model selection showed that the most parsimonious model of breeding arrival date retained the preceding moult date (implying covariation between life-cycle events) and the individual trait covariates age, breeding state (first-time versus experienced breeders), their interaction, and wean date as a pup as fixed effects (table 1). Arrival dates were positively correlated with wean date and the timing of the preceding moult. The positive linear trend with weaning date (slope = 1.26, 95% CI: 0.88–1.65) shows that females with earlier weaning dates as pups tended towards earlier breeding arrival dates as adults (figure 2*a*). Similarly, females that completed their moult and departed to sea earlier during the preceding moult haulout typically arrived earlier at the colony during the subsequent breeding season (slope = 1.92, 95% CI: 1.62–2.22) (figure 2*b*). Individual reaction norms were shifted to earlier or later dates compared with the population mean, but mostly followed the population slope, even though the likelihood ratio test of random slope with respect to moult date was significant (figure 3*a*). First-time breeders arrived earlier than experienced breeders of the same age, and experienced breeders tended to breed earlier at older ages (figure 4). None of the time-varying (environmental) covariates (long-term annual trend, population density or the climate indices SOI and SAM) explained a meaningful amount of variation in breeding season arrival dates (table 1). In other words, model fit, as measured by the deviance, did not improve sufficiently to infer that time-varying (environmental) covariates had any important effects. The most parsimonious model (described above) was considerably better supported by the data compared with the null model (table 1).

**Table 1.** Fixed-effect model selection for mixed model analysis of southern elephant seal breeding arrival timing at Marion Island. age: female age (all individuals aged 21 to 26 were considered to be 21); state: first- or experienced breeder (first-time and experienced breeders were separated at ages 4 to 7); moult: date of the last observation in the preceding moult haulout; wean: the individual's weaning date as a pup; SAM: Southern Annular Mode; SOI: Southern Oscillation Index; *N*: female breeding population size; trend: linear temporal trend. The number of parameters (n.p.), delta AIC and model deviance are given. All models contained a random year intercept and a random slope term for moult date in the female identity random effect. The most parsimonious model is in bold font.

fixed effects	n.p.	ΔAIC	deviance
**age** * **state + moult + wean**	**30**	**0**	**37483.20**
age * state + moult + wean + SOI + *N* + trend	35	4.30	37477.50
age * state + moult + wean + SAM + SOI + trend	35	4.73	37477.93
age * state + moult + wean + SAM + SOI + *N*	36	6.56	37477.76
age * state + moult + wean + SAM + *N* + trend	35	7.91	37481.11
age * state + moult + wean + SAM + SOI + *N* + trend	37	8.17	37477.37
age + moult + wean + SAM + SOI + *N* + trend	33	13.98	37491.18
state + moult + wean + SAM + SOI + *N* + trend	16	18.97	37530.17
moult + wean + SAM + SOI + *N* + trend	15	34.04	37547.24
age * state + moult + SAM + SOI + *N* + trend	36	47.42	37518.62
age * state + wean + SAM + SOI + *N* + trend	36	161.07	37632.27
intercept	6	325.72	37856.92

**Figure 5.**
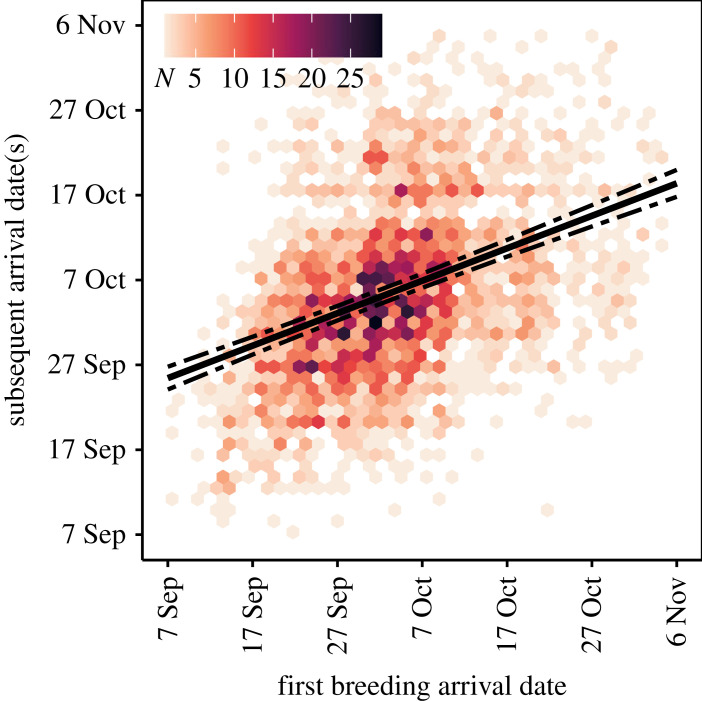
Within-individual repeatability in arrival date can be visualized by regressing individuals' arrival dates as experienced breeders against their arrival dates as a first-time breeder. The regression slope (solid line, with 95% CI as dashed lines; slope = 0.43, 95% CI: 0.39–0.47) shows that there is a significant correlation between first and subsequent arrival dates (i.e. that arrival date is repeatable within females). The distribution of the raw data is represented as a two-dimensional heat map of point counts per hexagon (*N*).

Between-individual variation contributed approximately 46% of the total variance in arrival dates estimated in the random effects structure of the mixed model ($\sigma_{i}^{2}=42.32$ [37.95–47.10]). This was approximately equal to the residual (within-individual) variance (48%; $\sigma_{\varepsilon }^{2}=44.56$ [42.08–46.66]) but substantially greater than the among-year variance (3.3%; $\sigma_{j}^{2}=3.08$ [1.67–5.55]). The covariance between the individual random effect intercept and the individual-by-moult date slope ($\sigma_{m}^{2}=2.58$ [1.20–4.19]) was low (cov. = 0.07). Variation in arrival date among females was therefore substantial, whereas annual variation contributed a small portion of the total variability in breeding arrival dates (figure 3*b*). Arrival dates for individual females ranged from 20 days before to 20 days after the population mean, though approximately 80% of females had a random effect intercept within 8 days of the population mean. The repeatability analyses confirmed important between-individual variation in the population, and that many individuals differed consistently from one another in their arrival date (*R* = 0.45 [0.42–0.48]) (figure 5). We calculated between-year repeatability in the same way to test if females consistently arrived earlier or later in some years compared with other years. The repeatability values for years were low (0.03 [0.02–0.05]), indicating low between-year variation in arrival dates relative to variation within years.

**Figure 6.**
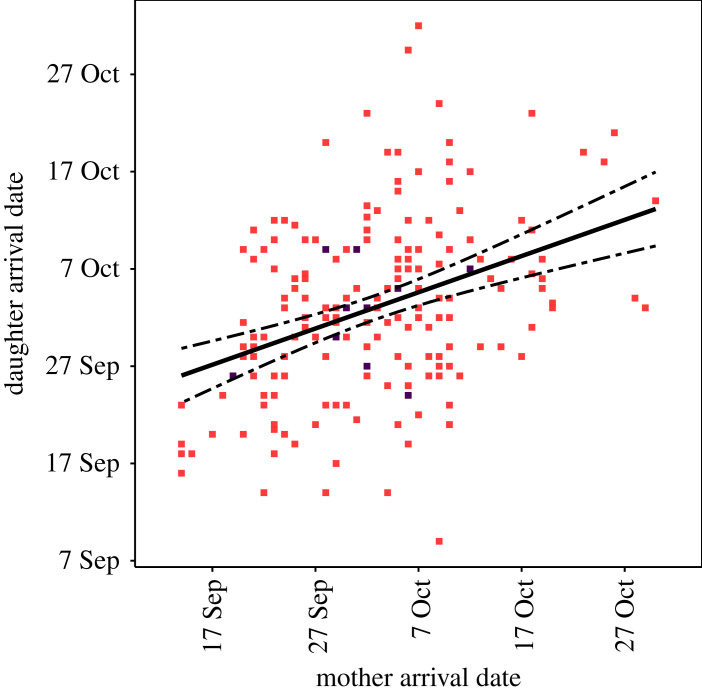
Regressing offspring phenotype on mother phenotype to estimate narrow-sense heritability (*h^2^*; here *h^2^* = 0.40). Red points are single data points; purple points are two overlapping observations. The positive slope shows a significant relationship between the first breeding arrival date of mothers and that of their daughters.

Parent–offspring regression analysis indicated that the arrival dates of daughters were significantly correlated to their mothers’ arrival dates (slope = 0.20, s.e. = 0.06, *p* = 0.001, *h*^2^ = 0.40) (figure 6). Daughters of earlier-breeding mothers thus also tended to have arrival dates that were earlier, on average, though the overall amount of variation explained by the parent–offspring regression was low (*R*^2^ = 0.05). Permutation tests showed that the predicted slope between arrival dates of random pairs of animals was mostly non-significant and centred around zero (mean slope = 0.01, 95% CI: −0.18–0.18), and that the observed parent–offspring regression slope coefficient was higher than expected by chance (one-tailed *p*-value < 0.01) (electronic supplementary material, figure S10).

## 4. Discussion

Our 30-year study of female elephant seal breeding phenology demonstrated five important points. It (1) highlighted a strong relationship between the timing of different parts of the annual cycle (moult and breeding phenology), (2) showed associations between breeding arrival dates and individual traits (wean date as a pup, age and breeding experience), (3) revealed mother–daughter similarities in breeding phenology, and (4) indicated high repeatabilities (i.e. individual consistency) of arrival date within females. We did not find evidence for (5) any strong relationship between breeding season arrival dates and measures of environmental variation (long-term trends, population density and climate indices).

Arrival dates were consistently early among young first-time breeding females, aligning with the findings of previous studies on elephant seals [25]. In contrast, whereas previous studies concluded that elephant seal females pup progressively later with age [20,24], we found evidence of earlier arrival among the oldest age classes. Arrival dates were latest for prime-age experienced breeders aged 4 to 8 years, while subsequent arrival dates became earlier with increasing age (from age 8, the linear regression slope of age was −0.41 (95% CI: −0.56 to −0.27; figure 4, electronic supplementary material, text S4). Our contrasting conclusion can be attributed to the absence (or low sample size) of old females in previous studies, and the analytical grouping of females into age-classes (e.g. all females over the age of 6 years were grouped together in [24]). Our results thus emphasize the value of utilizing long-term datasets containing information on individuals across the adult age spectrum. Southern elephant seal breeding arrival dates therefore exhibit a curvilinear pattern, with younger (primiparous) and older mothers arriving earlier in the season, which may help to establish priority positions in harems [24]. Curvilinear age-specific arrival patterns have also been reported for breeding Weddell seals (*Leptonychotes weddellii*) [55], grey seals (*Halichoerus grypus*) [56] and northern elephant seals (*Mirounga angustirostris*) [57]. In these species, however, both younger (primiparous) and older mothers breed later in the season, and not earlier as we show for southern elephant seals.

Elephant seal females time their pre-breeding migrations so that they return to the breeding colony a few days before birth [28,29]. Breeding arrival dates are thus chiefly determined by when implantation occurs, and the duration of gestation. Progressively earlier breeding with age may partly be explained by the links that exist between different parts of the annual cycle. Adult female elephant seals have approximately similar moult haulout start dates, but moult haulout duration becomes shorter with increasing age [33]. A shorter moult haulout duration means that female seals tend to complete their moult haulout slightly earlier as they reach old ages. Implantation of the blastocyst can occur before completion of the moult [31], but if departure from land and the resumption of foraging improve female physiological condition, this may influence the timing of implantation or rate of fetus development, and consequently the female's parturition date in the next breeding season.

Photoperiod is a major determinant of the reproductive cycles of seals, but other environmental cues and physiological condition would also determine when implantation occurs and embryonic growth commences, leading to annual variability in the timing of births [18]. The reaction norms of individual females revealed relatively large deviations from the population intercept and relatively low phenotypic plasticity (between-individual variance in slope) related to moult date. The population intercept is the average breeding arrival date of females at the average moult departure date of the population; the deviations from this line indicated that some females had consistently earlier or later breeding arrival dates relative to the population average. Approximately 46% of the total variation in arrival date was due to between-individual differences (deemed ‘personality traits’ in non-human behavioural ecology). The significant reaction norm slope term indicated that individuals differed in how they responded to changes in their moult departure dates, but the amount of variance explained by varying slopes was small (3%) relative to the variance explained by intercept adjustments. We do not know the cause of variation in arrival (and, we assume birth) dates among females, but variation in conception and implantation dates, or consistent differences in foraging success or gestation length could be potential drivers [58].

Parent–offspring regression showed that a significant proportion of the total variance in arrival dates was explained by additive genetic effects. Our permutation analysis, which drew dyads at random from the population, showed that the probability of obtaining a heritability estimate as high or higher than ours by chance was negligible. Establishing parent–offspring relationships in the field is not always easy, and our heritability analysis was therefore conducted on a subset of mother–pup pairs (paternity could not be established). Nevertheless, the significantly positive linear slope of the weaning date covariate in our main analysis supports the mother–daughter regression results. Pup weaning dates are related to the arrival date of a mother: mothers that arrive early in the breeding season have pups with earlier weaning dates, and these pups tend towards earlier breeding arrival dates as adults, plausibly because of a heritable component. Parent–offspring regression sometimes overestimates heritability of traits compared with analyses based on more complex pedigrees [59]. This may be because non-genetic maternal effects (any aspect of the mother's phenotype that affects offspring phenotype) or other environmental sources of covariance among parents and offspring that may inflate heritability estimations are not taken into account [59]. In this study, the arrival dates of daughters were obtained at least 3 years after the arrival dates of mothers, and as such, environmental sources of covariance are probably limited. Confounding non-genetic maternal effects are more probable and their presence would be an interesting finding in its own right. Alternatively, similarity in arrival dates of mothers and daughters could indicate reproductive timing is constrained by an annual cycle that takes a certain amount of time to complete, with an individual's own birth timing perhaps providing a starting point.

Our results describe, for the first time to our knowledge, how maternal genetic (or non-genetic) effects, individual traits and linkages between cyclical life-history events can drive within- and between-individual variation in reproductive phenology in elephant seals. By contrast, breeding arrival dates did not show a relationship with measures of environmental variation. Irrespective of the level of environmental variability, secondary consumers like elephant seals often show lower levels of phenological variability compared with primary producers and consumers [1]. Limited environmental adjustment of reproductive timing in Southern Ocean seals (elephant seals (this study) and Weddell seals [55]) contrasts with results from the Northern Hemisphere but has also been reported for some seabird populations [60]. Compared with seabirds, climate influences on marine mammal phenology are poorly represented in the literature, especially outside of the Arctic [61]. Nonetheless, as in seabirds, variable species- or region-specific patterns emerge. While the timing of breeding in southern elephant seals (this study) and Weddell seals [55] appears relatively consistent across years, the mean birth date of grey seals breeding in the Canadian Arctic advanced by 15 days between 1991 and 2017 [56]. Shifts in phenology have also been observed in other Northern Hemisphere seal populations (e.g. harbour seals, *Phoca vitulina* [62] and grey seals [63]). These shifts in breeding phenology of Northern Hemisphere seals are probably the result of changing environmental conditions that affect body condition, endocrine function, and the timing of implantation [64]. Marked between-year variation in timing of breeding (especially among resident species [60]) or long-term changes in breeding phenology have been reported in a range of seabird species [65,66]). Elephant seals and other long-distance migratory marine mammals (e.g. many whale species) provide an interesting comparison with more well-researched taxa such as migratory birds and temperate herbivores, which also exibit plasticity in the timing of migration and breeding [5,29]. Unlike income breeding species, such as insectivorous passerines, capital breeding female elephant seals do not forage during the breeding period. Local food availability during the breeding season is therefore irrelevant (cf. [11]), but females could rely on matching high post-breeding energy requirements to high availability of prey.

### (a) Limitations

We found no correlation between annual fluctuations in phenology and indices of environmental variation, but this does not mean that environmental controls of breeding phenology are absent. For example, the timing of breeding may be affected by unmeasured environmental conditions, or at a finer scale than we could detect. Indeed, for wide-ranging marine predators with extensive distributions, such as elephant seals, it is often challenging to obtain environmental covariates that accurately reflect changes in marine food webs and thus the availability of essentail resources (at the population and, especially, individual levels).

Our inferences were based on AIC model selection, which selects models based on their ability to reproduce the in-sample data (the data used to estimate the parameters of the model). The identified relationships therefore do not necessarily extend to causality [67]. Cause-and-effect analysis (e.g. structural equation modelling) may help to obtain a process-level understanding of the mechanisms that drive changes in phenology in this and other populations [68]. Furthermore, observational studies nearly always include some form of measurement error, which adds uncontrolled variation (noise) to predictions and increases uncertainty in parameter estimates [69]. In our case, weekly surveys of breeding sites lead to observed arrival dates that might be up to 6 days later than the actual arrival date. Failure to identify a female on the first survey for which it is present (electronic supplementary material, figure S2) will bias our estimate of arrival dates even more. Consequently, the observed data have an unknown bias towards later dates (a female can only be seen on her actual arrival date, or at a later date). Two predictor variables (weaning date and moult departure date) are subject to similar measurement errors. Noisy measurements combined with small sample sizes and small effect sizes may generate spurious statistical significance [69]. Our study had a large sample size (1755 females breeding 5167 times), giving us confidence that measurement error did not conceal important processes that underlie patterns in breeding phenology, even if the effect size (expected magnitude of phenotypic change) is relatively modest. A key assumption is that measurement error occurred completely at random with regards to predictor variables, and is therefore unlikely to produce misleading conclusions about the ecological hypotheses we posed. Despite the uncertainties that are inherent to the observational data we present in this study, we consider that our results reflect the ecological properties of the population.

We acknowledge that improved data or analysis methods can potentially increase confidence in our results. Alternative observation methods (e.g. passive integrated transponders or geolocation tags) or increased survey frequencies would allow a more accurate estimation of breeding arrival times in our population, but these methods are difficult to implement under current field conditions. Analytical approaches such as state–space models make it possible to model the true state of the system (e.g. arrival times) as a latent process, with the observed data conditional on this process and an observation error. Bayesian state–space models could potentially be used to incorporate an observation error distribution in one direction. Furthermore, site occupancy models [70,71] and nonparametric Bayesian capture–recapture models [72] have been developed to improve estimation of arrival times (e.g. arrival of the first individual at any site) where detection is not perfect. Further development of these models to fit our sampling design may give the most robust estimates of arrival times.

## 5. Conclusion

Our analysis of long-term individual-based data revealed that additive genetic effects, early-life traits, age and breeding experience, and the timing of other parts of the annual cycle influence the reproductive phenology of elephant seals. While many studies consider phenological shifts in animal life-history events in relation to changes in current environmental conditions, fewer consider how transgenerational effects, early-life events, demography and carry-over effects between cyclical life-history events may contribute to shape individual and population phenology distributions. Yet, population-level studies that do not account for these effects may reach misleading conclusions about environmental drivers of phenological change [65]. Much of our current knowledge of phenological change in vertebrates also comes from income breeding species, where selection favours synchronization of breeding and local food peaks (i.e. resource availability). The timing of reproduction is likely affected by different selective pressures in animals that are freed from this constraint (e.g. elephant seals and other capital breeders). An open remaining question is thus how the selective pressures and fitness consequences of phenological shifts differ across income and capital breeders.

## Data Availability

Data and R code can be found online at https://doi.org/10.5061/dryad.573n5tbd7 [73] and a live repository at https://github.com/ChrisOosthuizen/elephantsealPhenology.git. Supplementary material is available online [74].
